# Female genital mutilation/cutting experiences and attitudes among women from countries with high prevalence of FGM/C living in the United States: findings from the women’s health needs study

**DOI:** 10.1186/s12978-025-02244-2

**Published:** 2026-02-19

**Authors:** Margaret Christine Snead, Ekwutosi Okoroh, Ghenet Besera, Carrie K. Shapiro-Mendoza, Ashley N. Smoots, Petry Ubri, Roy Ahn, Vicki Pineau, Sabrina Avripas, Nicole Warren, Doris Mukangu, Crista E. Johnson-Agbakwu, Ayeesha Sayyad, Connie L. Bish, Howie Goldberg, Mary Goodwin, The WHNS Study Team, Madeleine Liotta, Ned English, Erin Fordyce, Manal Sidi, Farah Sublett, Maggie Cherney, Stephanie Alexander, Stephen Hayes, Yvonne Commodore-Mensah, Rihana Nesrudin, MamHarr Gaye, Rufo Jiru, Joey Dagher, Betselot Mekonnen, Zeinab Eyega, Consolatie Uwera, Hager Shawkat, Salwa Ahmed, Lilly Perry, Paul Stupp, Karen Pazol, Thomas A. Clark, Florina Serbanescu, Wanda Barfield, The WHNS Study Team

**Affiliations:** 1 Division of Reproductive Health, Centers for Disease Control and Prevention, Atlanta, GA USA; 2 Epidemic Intelligence Service, Centers for Disease Control and Prevention, Atlanta, GA USA; 3 Division of Violence Prevention, Centers for Disease Control and Prevention, Atlanta, GA USA; 4 NORC at the University of Chicago, Chicago, IL USA; 5 Johns Hopkins University School of Nursing, Baltimore, MD USA; 6 Amani Women Center, Atlanta, GA USA; 7 Office of Health Equity, Collaborative in Health Equity, The University of Massachusetts, Massachusetts, USA; 8 Obstetrics & Gynecology, UMass Memorial Health, Worcester, MA USA; 9 Division of Preventive and Behavioral Medicine, Population and Quantitative Health Sciences, University of Massachusetts T.H. Chan Medical School, Massachusetts, USA; 10 Independent Consultant, Atlanta, GA USA

**Keywords:** Female Genital Mutilation/Cutting, Female Circumcision, Migrant health, United States

## Abstract

**Background:**

Female genital mutilation/cutting (FGM/C) is a health and human rights concern for women and girls globally, and illegal to perform in the United States (US). FGM/C is associated with negative immediate and long-term health consequences such as pain, infections, and obstetric complications. Recent US estimates of the numbers of women and girls impacted by FGM/C are unknown but increasing immigration to the US from countries where FGM/C is prevalent suggests an increased population. We describe the health characteristics, experiences, and attitudes about FGM/C among Women's Health Needs Study (WHNS) participants.

**Methods:**

The WHNS cross-sectional survey interview collected information from 1,132 women ages 18 to 49 years living in the US who were born, or whose mothers were born, in a country where FGM/C is a prevalent practice. During November 2020 through June 2021, study participants were identified in four US metropolitan areas using a hybrid venue-based and respondent-driven sampling approach. We analyzed WHNS data to describe respondents’ characteristics, FGM/C experiences (FGM/C status, type of FGM/C, age at FGM/C, communication with health care providers about FGM/C) and attitudes about continuance of FGM/C. Analyses were conducted using SAS version 9.4.

**Results:**

Of the 1,132 women interviewed, over half (55%) had experienced FGM/C. Of those, 29% said their vagina had been sewn closed (infibulated), almost two-thirds (64%) reported that FGM/C occurred before age 10, and fewer than one third of women with FGM/C (31%) had ever discussed it with a health care provider. Most women interviewed thought FGM/C should be stopped (92%).

**Conclusions:**

Our results provide insights into FGM/C-related experiences and attitudes among 1,132 US women from FGM/C-practicing countries, among whom just over half reported having experienced FGM/C. Social and health care services that provide care to potentially affected US women can use this knowledge about women’s FGM/C experiences and attitudes to heighten awareness, improve clinical care, and promote interventions and strategies for prevention.

## Background

Female genital mutilation/cutting (FGM/C) is a health and human rights concern for women and girls globally. The World Health Organization (WHO) defines FGM/C as “all procedures involving partial or total removal of the external female genitalia or other injury to the female genital organs for non-medical reasons” [1]. FGM/C practice is concentrated in about 30 countries in Africa, Asia, and the Middle East [2], and is rooted in cultural and social traditions and beliefs such as rites of passage to adulthood, marriageability, honor, and preventing promiscuity [3, 4]. FGM/C has been associated with immediate and long-term negative health consequences for girls and women [5]. FGM/C is illegal to perform in the United States (US) [6, 7], and US government agencies [8] and professional medical organizations [9–11] have called for an end to the practice.

Numerous studies have described FGM/C experience and attitudes among women from countries with high prevalence of FGM/C who have migrated to the US [12–15], the European Union [16], England [17], and other high-income countries [18, 19]. In the US, immigration trends from countries where FGM/C is prevalent have contributed to an increase in the potential number of US women and girls who have experienced or been at risk of FGM/C [20]. Estimates for 2012 indicated that 513,000 women and girls residing in the US could have experienced or been at risk of FGM/C, tripling the estimated numbers published in the 1990 s (168,000 girls and women) [20, 21]. These US studies used indirect methods to estimate FGM/C and have limitations. For example, the estimates were derived using prevalence of FGM/C documented in national surveys in the countries of origin and assumed immigrants came from groups or communities who practiced FGM/C at the same national levels. Moreover, US national estimates based on more recent data are unavailable.

Some studies have collected information directly from US residents from FGM/C–practicing countries to document and understand attitudes and experiences surrounding FGM/C. A recent scoping review reported that US studies tended to focus on single countries of origin or communities, were limited by small sample sizes (< 100 respondents), and tended to overrepresent the most severe form of FGM/C due to the US studies’ focus on Somali women, among whom the most severe form of FGM/C predominates [22]. Studies that included women from multiple countries of origin found that the percentage of women with FGM/C varied by country [22]. Women with FGM/C reported having negative health care experiences in the US and had little communication with providers about their FGM/C [22]. Women and men from FGM/C-practicing countries living in the US commonly reported negative attitudes toward the practice [22].

CDC carried out WHNS to expand our understanding of the diverse FGM/C experiences and attitudes of US women and generate new information to help guide health care services and FGM/C prevention efforts.

## Methods

The Women's Health Needs Study (WHNS) study design, eligibility, instruments, translations, and implementation are detailed elsewhere [23]. Briefly, WHNS is a cross-sectional study implemented from November 2020 to June 2021 in four US metropolitan areas. The four areas were selected from the top 10 US metropolitan areas with concentrations of residents from 11 African countries [24] that had national FGM/C prevalence of at least 65% according to recent national surveys (Burkina Faso, Egypt, Eritrea, Ethiopia, Guinea, Mali, Mauritania, Sierra Leone, Somalia, Sudan, or The Gambia, hereafter called study “countries of origin”) [25]. The CDC’s Division of Reproductive Health (DRH) contracted with NORC at the University of Chicago and consulted with subject matter experts and community members to design and implement WHNS. In each study site, NORC collaborated with community-based organizations (CBOs) to implement WHNS.

We used a hybrid venue-based sampling (VBS) and a respondent-driven sampling (RDS) approach to identify eligible women. Both methods have previously been used in community-based studies that focused on stigmatized health issues [26–29]. WHNS participants were: 1) ages 18 to 49 years; 2) born in or had a mother born in a study country of origin, and 3) able to complete a telephone survey interview in one of the languages for which a translated questionnaire and interviewer were available (Amharic, Arabic, English, French, Oromo, Somali, or Tigrinya). CBOs recruited and interviewed VBS participants, who were then asked to recruit up to three potentially eligible RDS participants from their social network. Study population size goals for the numbers of completed survey interviews by country of origin and metropolitan area were based on the estimated size and composition of the populations in each of the four metropolitan areas according to the 2018 US Census Bureau American Community Survey (ACS) [24].

Questions about FGM/C experience, including type of FGM/C, were derived from the 2020 Demographic and Health Survey questionnaire [30] and previous FGM/C studies conducted in the US [31] and Europe [32]. Self-reported information from women about their family history of FGM/C and whether they had FGM/C was based on the following responses: *comes from a family that practiced FGM/C* (yes, no, don’t know, prefer not to answer); and *ever experienced FGM/C* (yes, no, don’t know, prefer not to answer). Among women who reported having experienced FGM/C, we used a hierarchical categorization of self-reported *FGM/C type* in the following order: genital area sewn closed (infibulated) (yes, no, don’t know, prefer not to answer), flesh removed from the genital area (yes, no, don’t know, prefer not to answer), nicked/no flesh removed from the genital area (yes, no, don’t know, prefer not to answer). We also included the respondent’s *attitude about continuation of FGM/C* (it should be stopped, it should continue as is, depends on the family, other/mixed feelings, don’t know, prefer not to answer). For the variable *FGM/C type*, “sewn closed” as a response option was the plain language terminology used in the survey and is used throughout the paper –which is referring to WHO terminology “infibulated” or “FGM/C type 3.” The variable *country of origin* grouped West African countries (Burkina Faso, Guinea, Mali, Mauritania, Sierra Leone, The Gambia) into a single regional category because the ACS did not separate out the West African countries included in our study (with the exception of Sierra Leone) [24]. The variable *immigration generation* classifications used in WHNS have been applied in previous immigration research and distinguish immigrants who arrived at ≥ 13 years of age (1.0 generation) from those who were < 13 years (1.5 generation) and therefore more likely to have had most of their education in the US, be fluent in English, and share some characteristics with US-born individuals whose parents were not born in the US (2.0 generation) [33]. All variables used in our analysis are described in detail in Supplemental Table 1.

**Table 1:** Respondent characteristics, Women’s Health Needs Study, 2020–21

**Characteristic**	**Total**	
	**N**^**a**^	**%**^**b**^
Total	1,132	100.0
Age (years)		
18–24	258	15.8
25–29	144	15.6
30–39	417	37.5
40–49	313	31.1
Country of origin^c^		
Egypt	59	11.1
Eritrea	137	2.7
Ethiopia	275	30.7
Somalia	173	13.3
Sudan	147	3.2
West Africa^d^	341	39.0
Highest level of education completed		
Less than a high school diploma	279	15.1
High school diploma/GED	306	22.7
Some college/Associate degree	322	32.5
Bachelor's degree or higher	225	29.7
Current marital status^e^		
Married/living with a partner	615	60.7
Previously married (widowed, divorced, separated)	164	14.4
Never married/lived with a partner	333	24.9
Number of children born alive^f^		
0	383	29.5
1–2	375	37.3
3–4	264	25.0
5 +	107	8.1
Health insurance^g^		
Private	333	30.5
Medicaid	588	52.5
No insurance	191	17.0
Length of time in the US^h^		
New arrivals (US immigration ≤ 5 years ago)	295	27.9
Established (US immigration > 5 years ago)	684	68.9
Born in US	149	3.2
Immigration generation^i^		
1.0 generation (Age of US immigration ≥ 13 years)	876	86.6
1.5 generation (Age of US immigration < 13 years)	101	10.1
2.0 generation (Born in US)	149	3.3
Comes from a family that practiced FGM/C^j^		
Yes	771	71.3
No	333	28.7

*FGM/C* Female Genital Mutilation/Cutting, *GED* General Education Development, *US* United States

^a^Unweighted *N*

^b^Weighted column percentage

^c^Country of origin is the participant's birth country (if born in a study country) OR mother's birth country (if participant was not born in a study country)

^d^Includes Burkina Faso, The Gambia, Guinea, Mali, Mauritania, and Sierra Leone

^e^Excludes 20 "Prefer not to answer" responses

^f^Excludes 1 "Don't know" and 2 "Prefer not to answer" responses

^g^Excludes 18 "Don't know" and 2 "Prefer not to answer" responses

^h^Excludes 3 missing and 1 "Prefer not to answer" responses

^i^Excludes 4 missing, 1 "Don't know" and 1 "Prefer not to answer" responses

^j^Excludes 24 "Don't know" and 4 "Prefer not to answer" responses

### Analysis

We conducted a descriptive analysis of selected respondent characteristics and FGM/C experiences and attitudes. We present unweighted counts and weighted percentages. We also present unweighted counts for “don’t know” and “prefer not to answer” responses and these responses are excluded from percentage calculations when they comprised 2.2% or less of the totals. Categorical variable frequencies and proportions were tested for independence using Pearson’s Chi-square test, with the level of significance set at *p* < 0.05. All analyses were performed using SAS 9.4.

### Weighting

Two types of weighting adjustments were applied to the survey data. First, we used information about respondents' self-reported network size (number of family and friends in the community) to create a mathematical model of the RDS recruitment process and weights to correct for unequal selection probabilities [34]. Second, we applied a post-stratification weighting adjustment for differences in age and country of origin distributions between the WHNS participants and the population of eligible women from the four metropolitan areas to reduce potential bias due to noncoverage, nonresponse, or other non-sampling errors [35]. The 2018 ACS was used for post-stratification weighting [24, 36]. Study estimates and measures of the precision of those estimates were constructed using the final weights that include both the RDS and post-stratification weighting adjustments.

### Institutional research approvals & verbal informed consent

Both the CDC and NORC Human Subjects Institutional Review Boards (IRBs) reviewed and approved the WHNS Human Subjects Protocol (CDC IRB Protocol 7123; NORC IRB Protocol 17.02.08 Project 8031/8630). Paperwork Reduction Act approval for data collection was received from the Office of Management and Budget (OMB # 0920–1264). Although no personally identifiable information was collected during the study process, the study covered sensitive topics (e.g., FGM/C) and received a Certificate of Confidentiality and a National Institute of Justice Privacy Certificate. WHNS interviewers obtained and documented verbal informed consent from potential participants in their preferred language.

## Results

### Respondent characteristics

Of the 1,132 women who completed a WHNS interview, 68.6% were 30 to 49 years of age (Table 1). More than one-third of respondents (39.0%) originated from a West African study country (Burkina Faso, The Gambia, Guinea, Mali, Mauritania, or Sierra Leone combined, hereafter called “West Africa”), 30.7% from Ethiopia, 13.3% from Somalia, 11.1% from Egypt, 3.2% from Sudan, and 2.7% from Eritrea. Almost two-thirds (62.2%) had attended some college or completed a college degree. Almost two-thirds of women (60.7%) were currently married or living with a partner. Less than a third (29.5%) had never had a live birth and 37.3% had 1 to 2 live births. Over half (52.5%) reported Medicaid as their type of health insurance coverage. Most WHNS respondents had immigrated to the US (96.8%); over two-thirds had immigrated more than 5 years ago (68.9%), and most were age 13 or older at immigration (1.0 generation) (86.6%). Almost three-quarters of women (71.3%) came from a family that had practiced FGM/C (Table 1).

### Experience of FGM/C

Over half of WHNS respondents (580 women; 55.0%) reported having experienced FGM/C (Table 2). FGM/C experience varied significantly by women’s age, country of origin, highest level of education completed, marital status, number of children born alive, length of time residing in the US, immigration generation, and family history of practicing FGM/C (all *p*-values < 0.0001). FGM/C was highest among women aged 40 to 49 (72.5%) and lowest among those aged 18 to 24 (25.6%) and ranged from 74.0% of women from Somalia to 36.0% of those from Egypt. More than three-quarters of women who had not attained a high school diploma reported FGM/C (77.1%), whereas under half of those with some college or an associate degree (48.8%) or with a bachelor’s degree or higher (48.5%) reported FGM/C.

**Table 2:** Female Genital Mutilation/Cutting (FGM/C) experience by Respondent Characteristics, Women's Health Needs Study, 2020–21 (*n* = 1,104^a^)

Characteristic	Experienced FGM/C				*P*-value^**d**^
	**Yes**		**No**		
	**N**^**b**^	**%**^**c**^	**N**^**b**^	**%**^**c**^	
Total	580	55.0	524	45.0	
Age					<.0001
18–24	36	25.6	218	74.4	
25–29	65	49.6	77	50.4	
30–39	249	55.5	159	44.5	
40–49	230	72.5	70	27.5	
Country of origin^e^					<.0001
Egypt	19	36.0	37	64.0	
Eritrea	72	62.2	54	37.8	
Ethiopia	132	46.1	135	53.9	
Somalia	116	74.0	57	26.0	
Sudan	52	51.2	94	48.8	
West Africa^f^	189	60.4	147	39.6	
Highest level of education completed					<.0001
Less than a high school diploma	203	77.1	72	22.9	
High school diploma/GED	154	57.1	140	42.9	
Some college/Associate degree	122	48.8	194	51.2	
Bachelor's degree or higher	101	48.5	118	51.5	
Current marital status^g^					<.0001
Married/living with a partner	392	61.9	208	38.1	
Previously married (widowed, divorced, separated)	109	65.0	49	35.0	
Never married/lived with a partner	69	34.2	258	67.6	
Number of children born alive^h^					<.0001
0	101	36.8	277	63.2	
1–2	206	52.7	157	47.3	
3–4	179	70.0	75	30.0	
5 +	94	90.1	12	9.9	
Health insurance^i^					0.6277
Private	146	53.2	178	46.8	
Medicaid	322	57.3	249	42.7	
No insurance	104	53.6	85	46.4	
Length of time in the US^j^					<.0001
New arrivals (US immigration ≤ 5 years ago)	160	50.7	128	49.3	
Established (US immigration > 5 years ago)	413	59.3	252	40.7	
Born in US	<10	7.0	141	93.0	
Immigration generation^k^					<.0001
1.0 generation (Age of US immigration ≥ 13)	536	58.7	315	41.3	
1.5 generation (Age of US immigration < 13)	36	39.9	65	60.1	
2.0 generation (Born in US)	<10	7.0	141	93.0	
Comes from a family that practiced FGM/C^l^					<.0001
Yes	543	72.1	211	27.9	
No	34	13.5	292	86.5	

*FGM/C* Female Genital Mutilation/Cutting, *GED* General Education Development, *US* United States

^a^Women who responded to FGM/C questions; excludes 21 "Don't know" and 7 "Prefer not to answer" responses

^b^Unweighted *N*

^c^Weighted row percentage

^d^Chi-square test

^e^Country of origin is the participant's birth country (if born in a study country) OR mother's birth country (if participant was not born in a study country)

^f^Includes Burkina Faso, The Gambia, Guinea, Mali, Mauritania, and Sierra Leone

^g^Excludes 19 “Prefer not to answer” responses

^h^Excludes 1 “Don’t know” and 2 “Prefer not to answer” responses

^i^Excludes 18 “Don’t know” and 2 “Prefer not to answer” responses

^j^Excludes 3 missing and 1 “Prefer not to answer” response

^k^Excludes 4 missing, 1 “Don’t know” and 1 “Prefer not to answer” responses

^l^Excludes 23 “Don’t know” and 1 “Prefer not to answer” responses

FGM/C was more common among currently and previously married women (61.9% and 65.0%, respectively) compared with never married women (34.2%). Ninety percent of women with five or more live-born children reported FGM/C compared with 36.8% of women with no live births. FGM/C was more frequent among women who had immigrated to the US more than 5 years ago (59.3%) compared with those who immigrated within the past 5 years (50.7%). A greater percentage of those who were 1.0 generation reported FGM/C (58.7%) compared with 1.5 generation women (39.9%). Seven percent of women born in the U S reported having FGM/C. The percentage of women with FGM/C was greater among women who came from a family that practiced FGM/C (72.1%) compared with those who did not (13.5%) (Table 2). When the analysis was restricted to women from a family that practiced FGM/C (*n* = 754), 76.1% of 1.0 generation women reported FGM/C compared with 52.6% of 1.5 generation women (data not shown in table). FGM/C did not vary significantly by women’s insurance status.

**Fig. 1:**
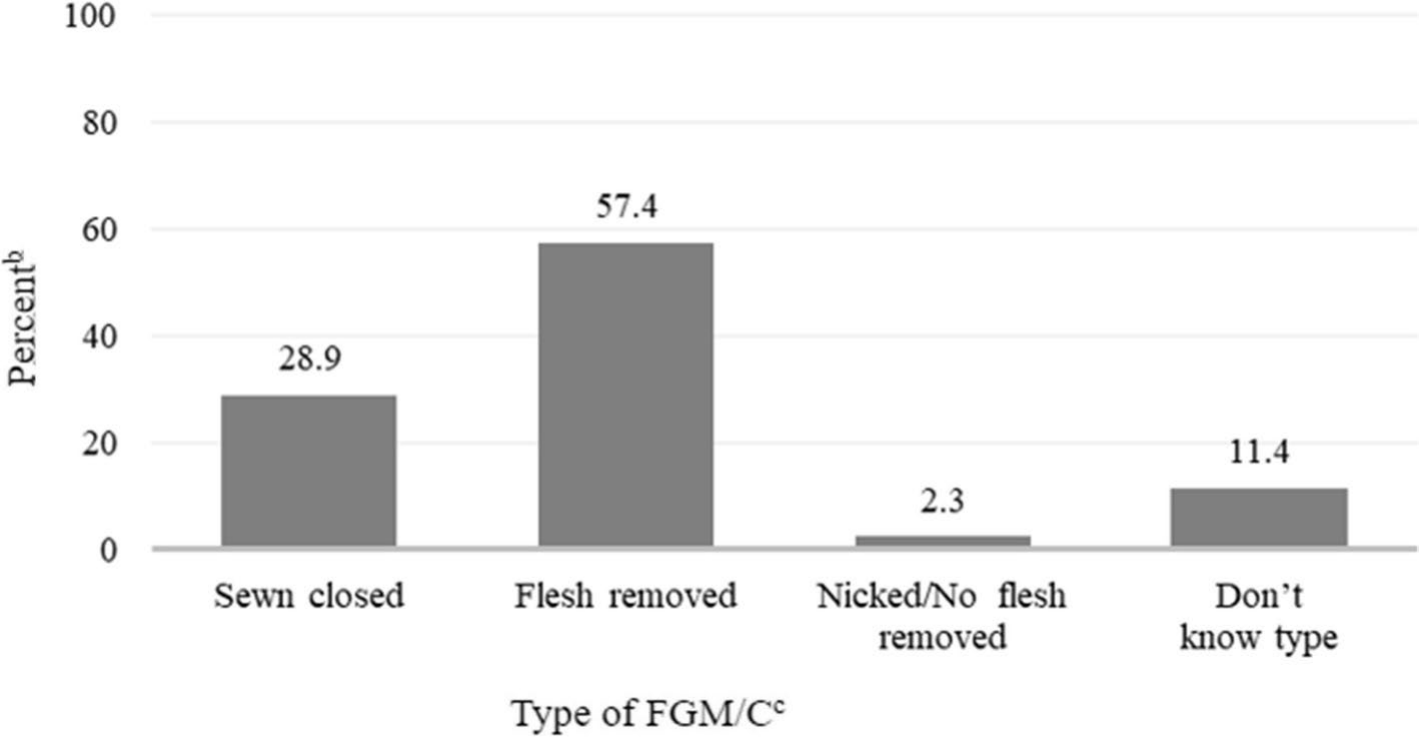
Type of Female Genital Mutilation/Cutting (FGM/C) Among Women Who Experienced FGM/C, Women's Health Needs Study, 2020–21 (*n* = 575^a^) FGM/C = Female Genital Mutilation/Cutting ^a^ Unweighted *N;* women with FGM/C who reported FGM/C type (*n* = 580), excluded 5 who responded "Prefer not to answer" ^b^ Weighted percentage ^c^ Classified as type of FGM/C experienced (self-reported) in the following order: sewn closed (infibulated), flesh removed, nicked/no flesh removed, don’t know type

Among 575 women with FGM/C who responded about the type of FGM/C they had, over half (57.4%) reported that they had flesh removed, 28.9% had been sewn closed (infibulated), 2.3% were nicked with no flesh removed, and 11.4% did not know what type of FGM/C they had (Fig. 1). Having been sewn closed (infibulated) was most reported by women from Sudan and Somalia (71.2% and 62.8%, respectively); having had flesh removed was most common among respondents who were from Ethiopia (71.8%), followed by respondents from West Africa (65.6%), and Eritrea (54.8%) (data not shown in figure).

**Fig. 2:**
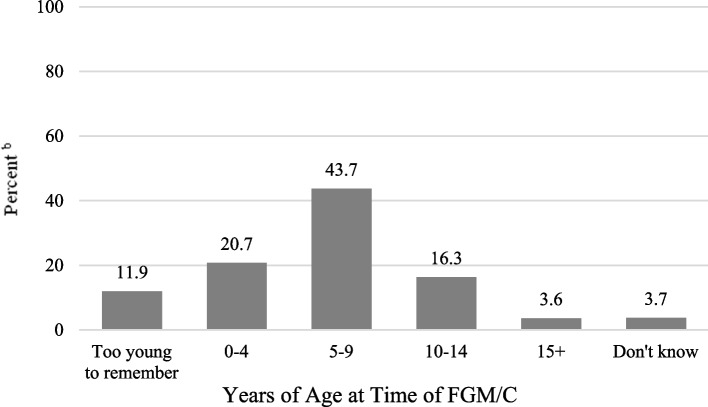
Age at Time of Female Genital Mutilation/Cutting (FGM/C) Among Women Who Experienced FGM/C: Women’s Health Needs Study, 2020–21 (*n =* 580^a^) FGM/C = Female Genital Mutilation/Cutting ^a^ Unweighted *N;* women with FGM/C who responded to question about age at FGM/C ^b^ Weighted percentages

The highest percentage of women reported having undergone FGM/C at 5 to 9 years of age (43.7%); 20.7% were 0 to 4 years old, 16.3% were 10 to 14 years old, and 11.9% said they were too young to remember when they underwent FGM/C (Fig. 2). Among women who were sewn closed (infibulated), 16.6% reported that they were 0 to 4 years old when they underwent FGM/C, 60.1% were 5 to 9 years old, 10.4% were 10 to 14 years old, 3.5% were age 15 and older, 7.7% were too young to remember and 1.7% did not know their age. Among those who had flesh removed, 21.0% reported that they were 0 to 4 years old, 40.2% were 5 to 9 years old, 20.7% were 10 to 14 years old, and 4.3% were age 15 and older, 9.6% were too young to remember and 4.2% did not know their age (data not shown in figure).

### Discussion with health care providers about FGM/C

Over half of women with FGM/C said that they would feel comfortable discussing FGM/C with a health care provider (57.5%), with significant variation seen by type of FGM/C (*p*-value = 0.0107) and country of origin (*p*-value < 0.0001) (Table 3). Among women who were sewn closed (infibulated), almost half (46.2%) said they would feel comfortable discussing their FGM/C with a health care provider compared with 64.0% who had flesh removed and 61.3% who did not know their FGM/C type. Feeling comfortable talking with a health care provider about FGM/C ranged from a high of 80.6% among women from West Africa to a low of 35.4% among women from Somalia.

**Table 3:** Comfort and experience Discussing Female Genital Mutilation/Cutting (FGM/C) with a Health Care Provider among Women who Experienced FGM/C: Women’s Health Needs Study, 2020–21 (*n* = 580^a^)

Characteristic	Would feel comfortable discussing their FGM/C with a health care provider				Ever discussed their FGM/C with a health care provider			
	**N**^**a**^	**Yes** **%**^**b**^	**No** **%**^**b**^	***P*-value**^**c**^	**N**^**a**^	**Yes** **%**^**b**^	**No** **%**^**b**^	***P*-value**^**c**^
Total	580	57.5	42.5		580	30.9	69.1	
Type of FGM/C ^d^				0.0107				0.3781
Sewn closed	191	46.2	53.8		191	34.5	65.5	
Flesh removed	301	64.0	36.0		301	32.2	67.8	
Nicked/no flesh removed	18	*	*		18	*	*	
Do not know type	65	61.3	38.7		65	22.4	77.6	
Age, years				0.0780				0.0003
18–24	36	44.5	55.5		36	2.4	95.6	
25–29	65	72.2	27.8		65	39.2	60.8	
30–39	249	53.9	46.1		249	25.4	74.6	
40–49	230	58.1	41.9		230	38.4	61.6	
Country of Origin^e^				<.0001				<.0001
Egypt	19	*	*		19	*	*	
Eritrea	72	52.1	47.9		72	14.7	85.3	
Ethiopia	132	48.2	51.8		132	17.2	82.8	
Somalia	116	35.4	64.6		116	17.2	82.8	
Sudan	52	51.7	48.3		52	31.2	68.8	
West Africa^f^	189	80.6	19.4		189	49.9	50.1	

*FGM/C* Female Genital Mutilation/Cutting

^a^Unweighted *N;* Women with FGM/C who responded to questions about health care provider communication

^b^Weighted row percentage

^c^Chi-square test

^d^Classified as type of FGM/C experienced (self-reported) in the following order: sewn closed (infibulated), flesh removed, nicked/no flesh removed, don't know type (excludes 5 women who didn’t answer)

^e^Country of origin is the participant's birth country (if born in a study country) OR mother's birth country (if participant was not born in a study country)

^f^Includes Burkina Faso, The Gambia, Guinea, Mali, Mauritania, and Sierra Leone

^*^ < 25 cases; not included in Chi-square test

Just under one-third of women with FGM/C (30.9%) had ever discussed their FGM/C with a health care provider (Table 3), and this varied significantly by age (*p*-value = 0.0003) and country of origin (*p*-value < 0.0001). More than one third of women aged 25 to 29 and 40 to 49 had spoken with a health care provider about their FGM/C (39.2% and 38.4%, respectively), as had half of women from West Africa (50.1%). In contrast, 17.2% of those from Somalia and Ethiopia, and 14.2% from Eritrea had ever spoken about their FGM/C with a health care provider. Among women who had never discussed their FGM/C with a health care provider, most said they would not feel comfortable doing so (88.7%) (data not shown in table).

### Attitude about Whether FGM/C should continue

Most survey respondents (both with/without FGM/C) said that FGM/C should be stopped (92.0%). Attitude about continuance of FGM/C varied significantly by the woman’s personal experience of FGM/C (*p*-value = 0.0013), type of FGM/C (*p*-value < 0.0001), and country of origin (*p*-value = 0.0032). Those with personal experience of FGM/C were less likely to say that FGM/C should be stopped (88.7%) compared with those without FGM/C experience (95.7%) (*p*-value = 0.0013). Among women with FGM/C, the percent who said it should be stopped was highest among those who were sewn closed (infibulated) (92.0%) or had flesh removed (91.5%) and lowest among women who did not know their type of FGM/C (72.0%) (Table 4). The percent who said that FGM/C should be stopped ranged between 95.7% among women from Ethiopia to 83.2% of women from Somalia. Among WHNS respondents who had immigrated to the US (*n* = 979), 24.7% said that their opinion about FGM/C had changed since immigration; almost all (98.4%) said they had become less accepting of the practice after immigrating (data not shown in table).

**Table 4:** Attitude about whether FGM/C should continue: Women’s Health Needs Study, 2020–21 (*N* = 1,115^a^)

**Characteristic**		**Attitude about whether** **FGM/C should continue**				***P*-value**^**c**^
	**N **^**a**^	Should be stopped **%**^**b**^	Should continue as is **%**^**b**^	Depends on the family **%**^**b**^	Other/mixed feelings **%**^**b**^	
Total	1,115	92.0	0.7	4.7	2.5	
Experienced FGM/C						0.0013
Yes	575	88.7	1.2	6.4	3.8	
No	514	95.7	0.2	3.0	1.1	
Type of FGM/C ^d,e^						< 0.0001
Sewn closed	190	92.0	0.0	4.4	3.6	
Flesh removed	298	91.5	1.1	3.6	3.8	
Nicked/no flesh removed	18	*	*	*	*	
Don't know type	64	72.0	3.8	20.2	4.0	
No FGM/C	540	95.7	0.2	3.0	1.1	
Age, years						0.3350
18–24	250	87.7	1.5	8.2	2.5	
25–29	143	92.2	0.0	5.4	2.4	
30–39	414	93.4	0.3	4.6	1.7	
40–49	308	92.4	1.2	2.8	3.6	
Country of origin^f^						0.0032
Egypt	58	91.9	0.0	1.2	6.8	
Eritrea	135	89.3	1.5	5.9	3.3	
Ethiopia	272	95.7	0.4	2.8	1.1	
Somalia	173	83.2	0.3	13.8	2.7	
Sudan	143	94.0	0.2	2.4	3.5	
West Africa^g^	334	92.2	1.3	4.2	2.2	
Highest level of education completed						0.2073
Less than a high school diploma	276	87.6	1.4	7.0	4.0	
High school diploma/GED	301	90.7	0.5	6.7	2.1	
Some college/Associate degree	318	93.9	1.1	2.8	2.2	
Bachelor’s degree or higher	220	93.2	0.0	4.2	2.5	
Length of time in the US^h^						0.4851
New arrivals (US immigration ≤ 5 years ago)	291	93.6	0.0	5.5	0.9	
Established (US immigration > 5 years ago)	676	91.5	1.0	4.4	3.1	
Born in US	144	92.9	0.2	0.9	6.0	
Immigration generation^i^						0.0512
1.0 generation (Age of US immigration ≥ 13)	866	92.8	0.5	4.1	2.6	
1.5 generation (Age of US immigration < 13)	99	85.9	2.3	10.3	1.5	
2.0 generation (Born in US)	144	92.9	0.2	0.9	6.0	
Comes from a family that practiced FGM/C^j^						0.6271
Yes	761	91.8	0.6	5.0	2.6	
No	330	93.0	1.1	4.4	1.6	

*FGM/C* Female Genital Mutilation/Cutting

^a^Unweighted *N; w*omen who responded to question about attitude towards FGM/C, excluding 15 "Don't know" and 2 "Prefer not to answer" responses

^b^Weighted row percentage

^c^Chi-square test

^d^Classified as type of FGM/C experienced (self-reported) in the following order: sewn closed (infibulated), flesh removed, nicked/no flesh removed, don't know type

^e^Excludes 5 women with FGM/C who "Prefer not to answer"

^f^Country of origin is the participant's birth country (if born in a study country) OR mother's birth country (if participant was not born in a study country)

^g^Includes Burkina Faso, The Gambia, Guinea, Mali, Mauritania, and Sierra Leone

^h^Excludes 3 missing and 1 “Prefer not to answer” response

^i^Excludes 4 missing, 1 “Don’t know” and 1 “Prefer not to answer” responses

^j^Excludes 23 “Don’t know” and 1 “Prefer not to answer” responses

^*^ < 25 cases; not included in Chi-square test

#### Discussion

Our analysis expands understanding of the FGM/C-related experiences and attitudes of study participants in four metropolitan areas in the US who were born, or whose mothers were born, in a country where FGM/C is a prevalent practice. Just over half of WHNS respondents reported having experienced FGM/C, with variations by age, country of origin, and other individual characteristics. We found that a smaller proportion of women in the youngest age group reported FGM/C compared with women in the oldest age group. This is consistent with recent population-based surveys in Africa that have found lower FGM/C prevalence in younger age cohorts (e.g., 18 to 24 years of age) compared with older age cohorts (e.g., 40 to 49 years of age), suggesting the practice of FGM/C has declined over time [3]. We also found that the percentage of WHNS respondents who reported FGM/C varied by country of origin. This is consistent with both comparative analyses of global surveys [3] and previous US studies [12, 14, 37, 38] that included women from multiple African countries. Our findings about how FGM/C experience varies by age and country may help US social service and health care providers better understand women and girls affected by FGM/C. We found the predominant type of FGM/C among study respondents was flesh removal, which was highest among women from West Africa, Ethiopia, and Eritrea. Previous US research has focused primarily on Somali populations who were more likely to have been sewn closed (infibulated) [22], consistent with our findings.

Our study did not ask respondents with FGM/C in which country they experienced it, but we found that most (76.3%) women reported that they were younger than ten years of age at the time of FGM/C. FGM/C was highest (58.7%) among those who immigrated at ages 13 and older, and few (7%) US-born respondents reported FGM/C, suggesting that most FGM/C occurred before arrival in the US. These results are consistent with findings from the Netherlands [39] where women born in the Netherlands to mothers who had immigrated from countries with high prevalence of FGM/C had much lower risk of FGM/C compared with women of similar ages who immigrated to Netherlands after birth from a high-prevalence country. Although few US-born women in our study reported having FGM/C, it is illegal under US law to perform FGM/C on anyone under age 18, whether it is carried out in the US or during foreign travel [6, 7].

In our study, while over half the women with FGM/C reported they would feel comfortable discussing FGM/C with a health care provider, less than one third had done so. Most women with FGM/C who had never discussed FGM/C with a health care provider said they would feel uncomfortable doing so. Although WHNS did not ask women why they felt uncomfortable discussing their FGM/C with a health care provider, in other US studies women have reported embarrassment [40], fear of reprisal [40], and perceptions that health care providers lacked understanding of FGM/C practices [41] and reasons [22].

Finally, almost all (92%) of the women interviewed reported that FGM/C should be stopped, a result similar to findings from a scoping review of US studies [22]. Almost all women whose opinion of FGM/C had changed since immigration had become less accepting of the practice, which is also consistent with previous US research [14, 42, 43]. Attitudes opposing FGM/C have also increased over time in high-prevalence countries of origin, signaling a global trend toward less acceptance [44].

This analysis advances US research about FGM/C in several important ways. WHNS was a large US study that collected information from more than 1,000 women who were born or whose mothers were born in diverse countries of origin with high prevalence of FGM/C. Because of the size and diversity of the study population, we were able to examine experiences and attitudes by age group, country of origin, type of FGM/C, immigrant generation, and other demographic characteristics.

WHNS has some limitations. The study population was limited to women in four metropolitan areas; therefore, findings cannot be generalized to women in other areas of the US. Moreover, the percentage of women who reported FGM/C should not be interpreted as a prevalence estimate. The study population is more reflective of foreign-born women (1.0 and 1.5 generation) than of women born in the US whose mothers were born in another country (2.0 generation). There may be unknown biases due to demographic differences in populations who immigrate to the US compared with those who do not. Also, some otherwise eligible participants may have been excluded due to lack of study material language translations in their preferred language. The translated materials cover the main language-groups in the countries surveyed, but translations into less common languages, dialects, or tribal languages were not available. Interpretation of our findings may be limited by coverage error, nonresponse, and/or self-selection biases [45]. Given that FGM/C is a private, sensitive topic and that FGM/C is illegal in the US, information about FGM/C may have been underreported [46–48]. For FGM/C type, we relied on three descriptive questions that asked if flesh had been removed from the genital area, if the genital area had been nicked without flesh removal, or if the genital area had been sewn closed (infibulated) [23]. Women who answered these questions may not have accurately reported their type of FGM/C and 11% of participants reported that they did not know their FGM/C type. WHNS did not include confirmation of FGM/C status through a clinical examination. Some studies that have compared self-reported FGM/C with the results of clinical examinations found that although women did report reliably whether they had FGM/C or not [46–49], those with FGM/C tended to underreport the extent of their FGM/C type [46, 47, 49].

Some countries known to practice FGM/C were not considered for inclusion in WHNS or our analysis, either because the US population originating from these countries is relatively small or because available evidence comes from small-scale studies and there are no representative data on FGM/C prevalence among women 18 to 49 years of age [3]. Using national prevalence of FGM/C as the basis for country selection excluded some countries with lower national prevalence, but higher sub-national prevalence (e.g., Kenya, Nigeria) [3]. Our analysis did not account for sub-national ethnic, cultural, or geographic origins known to influence FGM/C practice within countries [3].

### Implications for prevention efforts and programs

Global abandonment of FGM/C is an objective of the United Nations (UN) [50] and is included in the 2030 UN Sustainable Development Goal 5.3 [51]. In May 2023, the U S’ White House Gender Policy Council released the first-ever US National Plan to End Gender-Based Violence [52], a plan that includes FGM/C as a form of gender-based violence to be eliminated. US professional medical organizations including the American College of Nurse Midwives [9], the American College of Obstetricians and Gynecologists [10], and the American Medical Association [11], have called for an end to FGM/C, as has a broad coalition of individuals and organizations affiliated with the US End FGM/C Network [53].

Our findings can inform prevention efforts and programs aimed at abondoning FGM/C. Primary prevention efforts to end its practice are often carried out through multifaceted legal, socio-economic, behavioral, and cultural interventions [54]. Primary prevention may be most effectively integrated into community-based programs already serving affected populations [55], including health education and community-level dialogues with parents, communities, and religious leaders [55]. Our results indicate a potentially large base of support for discontinuation of FGM/C among US women from high-prevalence countries, including those women who experienced FGM/C. Perspectives of women who have undergone FGM/C and who publicly oppose the practice have been made available by advocacy groups in videos and podcasts to inform prevention and intervention strategies for the practice [56].

Both secondary and tertiary prevention are important to ensure that women affected by FGM/C receive care fromproviders prepared to address this sensitive issue and deliver evidence-based services that reduce long-term consequences [57, 58]. Our findings highlight the need for more open and supportive dialogue, with greater use of person-centered communication and trauma-informed care. Screening, such as optional questions on maternity admission forms or in clinical or community settings, can help identify affected women but should only be used when responses will meaningfully improve care. 

Yet screening is not routinely offered, limiting access to prevention and treatment. Without it, women may miss opportunities for support with FGM/C abandonment or care to address complications. Evidence-based interventions, including defibulation and therapies for emotional and reproductive health, can help reduce long-term harm [59].

A survey of US health care provider attitudes and experiences found that fewer than half of respondents had received any formal training about FGM/C, and that almost 80% wanted more education on the topic [60]. Numerous tools to educate clinicians about FGM/C, its potential health implications, and culturally competent care for women with FGM/C are available in the US [61–63]. Increased knowledge about FGM/C and competency-based education are needed to provide high quality care to girls and women affected by FGM/C and to prevent the practice from being performed on girls who might be at risk [64].

Future studies of US women potentially affected by FGM/C could be expanded to include other US geographic areas and additional related topics. For example, studies might examine information on the impact of acculturation, cultural and social influences that shape changing attitudes toward FGM/C, experiences of intimate partner violence and other traumatic experiences, and mental health. The perspectives of girls, men, extended family members, and community leaders could provide a more well-rounded understanding of FGM/C in US communities. Finally, future research could evaluate how to implement what other countries (such as Australia, Canada, and United Kingdom) have been addressing: establishing centers or units within hospitals that specialize in the healthcare of FGM/C-affected women, ways to improve knowledge/communication/the quality of healthcare for FGM/C impacted women, educating healthcare professionals, and ways that the women in this study can become advocates for abandonment of FGM/C practice for their community through existing organizations in the US.

#### Conclusion

Our analysis expands understanding of the diverse FGM/C-related experiences and attitudes of 1,132 US women from FGM/C-practicing countries. WHNS found that slightly more than half of study participants had FGM/C. Among those who had FGM/C, more than one-quarter reported having been sewn closed (infibulated), and nearly two-thirds reported having FGM/C before 10 years of age. Almost all women, including those who had experienced FGM/C, expressed that FGM/C should be discontinued. Findings from our analysis to were disseminated nationally and at the community level through publications, factsheets in the study languages, and webinars to inform educational and behavioral interventions involved in the abandonment of FGM/C and to address the health of women who have experienced it. Health care and social services that provide care to potentially affected US women from FGM/C-practicing countries can use this knowledge about women’s FGM/C experiences and attitudes to heighten awareness, improve clinical care, and promote interventions and strategies for prevention.

## Data Availability

Data sets were generated and analyzed. Due to the sensitive nature of the subject matter they are not available.
